# Evaluation of internet derived patient information

**DOI:** 10.1308/10.1308/003588412X13171221590250

**Published:** 2012-07

**Authors:** JBM Ward, P Leach

**Affiliations:** ^1^Cardiff University,UK; ^2^Cardiff and Vale University Health Board,UK

**Keywords:** Hydrocephalus, Patients, Internet

## Abstract

**INTRODUCTION:**

The internet is a widely used, powerful resource for patients to research medical conditions. There is an extensive amount of information available on the internet. It is important for patient information to be accurate and in an easily accessible format. This article aims to assess the quality of patient information on hydrocephalus and compares the findings with recent evaluations in other surgical specialties.

**METHODS:**

The term ‘hydrocephalus’ was searched for on the search engines http://www.google.com/, http://www.bing.com/ and http://www.yahoo.com/. The top 20 results of these searches were assessed using the University of Michigan consumer health website evaluation checklist.

**RESULTS:**

The quality of patient information websites on hydrocephalus is highly variable. Websites rarely provide sufficient authorship information, do not review their information regularly enough and only reference material occasionally. The background of the provider was found to influence the quality of the website, with academic and care providers creating the best websites.

**CONCLUSIONS:**

On comparing our findings with those of recent studies from other surgical specialties, it was found that there was often a conflict of interest between the background of the provider and the information supplied. It is recommended that clinicians personally research material for their patients to be able to guide them to suitable, accurate websites.

The internet is a valuable and popular research tool for patients to find out more about their medical illnesses. The internet is widely accessible to the general population with 77% of people in Great Britain in 2011 having access at home.^1^ A third of patients are reported to look up medical information on the internet^2^ and 11% of patients research their symptoms prior to an outpatient appointment.^3^

Patients use the internet for many reasons related to their health. They may explore their symptoms prior to a consultation, develop further questions for the clinician, gain knowledge about a procedure or find support groups.^2^ This dependence on a widely unregulated resource provides numerous challenges to the health community with potential for patients to develop mistaken, potentially dangerous ideas about their health. It is important for the information provided by websites to be set at an appropriate level of understanding for the audience and to provide accurate, unbiased information that complements material provided by the clinician. The information provided on a website should be as accurate as in any other publication. The publisher should strive to achieve this as users are not always aware how to check a website’s credibility.

## Methods

The quality of patient information on hydrocephalus available on the internet was evaluated using three main search engines: http://www.google.com/, http://www.bing.com/ and http://www.yahoo.com/. The top 20 results from these searches were assessed using the University of Michigan consumer health website evaluation checklist.^4^

## Results

A total of 24 websites were found for evaluation. Some websites gave a very basic summary while others gave more detailed medical information aimed at health professionals. It seems likely that a patient’s previous level of medical understanding will influence his or her ability to assess a site. Very few websites provided links to support groups and those that did were often not relevant to supporting patients with hydrocephalus.

Websites described the authorship of their information poorly. They often omitted the author’s personal details, their relevance to the topic and contact details. Only half of the websites gave the date on which content was last reviewed. Few websites provided references. Several sites had numerous distracting animated adverts that diverted attention away from the informational content.

The type of website provider influenced the quality of the site. Academic and care providers created the highest scoring websites while charities and news agencies produced the lowest.

## Discussion

In an investigation into oesophageal cancer information, Reid *et al* found few websites providing authorship, date of last review and references.^5^ The accuracy of information about prognosis was investigated; most websites did not provide a numerical estimate and those that did were too vague.

Killeen *et al* investigated gastric cancer patient information and found that sites were overly commercial, the information provided was incomplete and the websites were often difficult to access.^6^ Moran and Oliver assessed internet information on hip fractures and frequently found a conflict of interest between the provider and the information available.^7^ They also found similar issues of poor recognition of authorship and referencing.

Muthukumarasamy *et al* evaluated thyroidectomy patient information using the Lida website validation method.^8^ They found the quality of websites highly variable and that the rank of the site within the search was not a good indicator of the site’s quality. When investigating cervical spine disc herniation, Morr *et al* also found that information was insufficient and that a large proportion of websites were physician sponsored.^9^ Finally, Aldairy *et al* used the DISCERN instrument to assess websites providing information on orthognathic surgery.^10^ They generally found further development was required on the websites due to the poor quality of information provided.

## Conclusions

Despite recommendations for improvement in the publications noted above, there has been limited progress in the past five years in the quality and accuracy of information delivered by websites. There is often a conflict of interest as many websites are provided by commercial companies. In order to improve the quality of patient information available on the internet, the following recommendations are offered to website providers:

- State the author’s details and contact information clearly.
- Reference material accurately and state when the site was last reviewed.
- State any conflict of interest.
- Any adverts should not be animated, flash or distract from the content of the website.
- Language should be plain, easily understood and with a minimal amount of medical jargon.

Surgeons should be aware that the****quality of patient information available on the internet is highly variable and that a conflict of interest is often present. It is therefore recommended that clinicians research websites so that they can guide patients towards reliable websites set at an appropriate level to that individual.
